# Prevention of cognitive decline and dementia: Current evidence on lifestyle factors and dietary patterns

**DOI:** 10.17179/excli2026-9298

**Published:** 2026-05-27

**Authors:** Ligia J. Dominguez, Nicola Veronese, Francesco Saverio Ragusa, Flavia Seminara, Salvatore Maria Baio, Laura Vernuccio, Giuseppina Catanese, Mario Barbagallo

**Affiliations:** 1 Sport and Exercise Sciences Research Unit, Department of Psychology, Educational Science and Human Movement, University of Palermo, 90133 Palermo, Italy; 2 Geriatric Unit, Department of Internal Medicine and Geriatrics, University of Palermo, 90100 Palermo, Italy; 3 Faculty of Medicine, Saint Camillus International University of Health Sciences, 00131 Rome, Italy

**Keywords:** dementia, cognitive decline, social engagement, sleep, exercise, dietary pattern

## Abstract

Cognitive decline and dementia represent major and growing global health challenges, driven largely by population aging and increased longevity. Currently, more than 55 million people worldwide live with dementia, a figure projected to rise to approximately 153 million by 2050. Alzheimer's disease accounts for the majority of cases, and despite extensive research, effective disease-modifying therapies remain limited. Consequently, increasing attention has shifted toward prevention strategies targeting modifiable risk factors. Accumulating evidence indicates that dementia is not an inevitable consequence of aging and that up to 45 % of cases may be attributable to potentially modifiable lifestyle and environmental factors operating across the life course. Lifestyle behaviors-including diet, physical activity, smoking, alcohol consumption, sleep, and social and cognitive engagement-have emerged as key targets for intervention. In particular, adherence to healthy dietary patterns such as the Mediterranean, DASH, and MIND diets has been associated with better cognitive outcomes, while unhealthy dietary patterns may increase risk. However, findings across studies remain heterogeneous, and uncertainties persist regarding causality, optimal exposure timing, and specific lifestyle components. Recent large prospective cohorts, meta-analyses, umbrella reviews, and multidomain intervention trials have advanced understanding of these associations but have also highlighted important gaps, including limited randomized evidence and underrepresentation of diverse populations. This narrative review critically synthesizes current evidence on lifestyle factors and dietary patterns associated with cognitive decline and dementia risk, focusing on recent high-quality studies. By integrating findings across domains, it aims to clarify areas of consensus and uncertainty, inform prevention strategies, and identify priorities for future research and public health action.

See also the graphical abstract(Fig. 1).

## 1. Introduction

Cognitive decline and dementia constitute growing global health challenges, with profound implications for individuals, families, healthcare systems, and societies (The Lancet, 2025[114]). Worldwide, the number of people living with dementia is projected to increase substantially over the coming decades, largely driven by population aging and improved survival (GBD 2019 Dementia Forecasting Collaborators, 2022[35]). The World Health Organization estimates that more than 55 million people worldwide live with dementia, with around 10 million new cases each year (World Health Organization, 2019[129]). Alzheimer's disease is the leading cause, responsible for 60-80 % of cases, followed by vascular dementia and other subtypes (Reuben et al., 2024[95]). The global burden of dementia is projected to rise dramatically, reaching approximately 153 million people by 2050 (GBD 2019 Dementia Forecasting Collaborators, 2022[35]). Dementia is a leading cause of disability (Vargese et al., 2023[121]), and no curative treatments currently exist (Fox et al., 2025[30]). Consequently, identifying modifiable risk factors is essential for delaying or preventing disease onset, as growing evidence indicates that a substantial proportion of dementia cases are linked to lifestyle and environmental factors that are modifiable across the life course (Jones et al., 2024[53]; Livingston et al., 2024[68]).

Despite extensive research efforts, disease-modifying therapies remain limited, and current treatments primarily offer symptomatic relief (Fox et al., 2025[30]; Frisoni et al., 2025[31]). There is increasing emphasis on prevention strategies aimed at delaying onset, slowing progression, or reducing overall risk at the population level that has been summarized in the Lancet Commission on Dementia Prevention, Intervention, and Care reports (Livingston et al., 2024[68]; Livingston et al., 2020[69]; Livingston et al., 2017[70]).

A substantial body of epidemiological evidence indicates that dementia is not an inevitable consequence of aging (Dominguez et al., 2021[22]). Rather, it is influenced by a complex interplay of genetic, biological, environmental, and lifestyle factors operating across the life course (World Health Organization, 2019[129]). Recent estimates suggest that up to 45 % of dementia cases may be attributable to potentially modifiable risk factors, highlighting significant opportunities for prevention (Livingston et al., 2024[68]). Modifiable lifestyle behaviors such as social engagement, sleep patterns, smoking, alcohol consumption, physical activity, and diet have emerged as central targets for intervention because of their broad impact on overall health. (Dominguez et al., 2021[22]).

Dietary patterns have received particular attention given their influence on cardiometabolic health, inflammation, oxidative stress, and neurovascular integrity, all of which are implicated in neurodegeneration (Wen et al., 2025[126]). Adherence to healthy dietary patterns such as the Mediterranean (MeDiet) (Fekete et al., 2025[28]; Wang et al., 2025[124]), Dietary Approaches to Stop Hypertension (DASH) (Song et al., 2024[111]), and Mediterranean-DASH Intervention for Neurodegenerative Delay (MIND) (Wang et al., 2025[124]; Chen et al., 2023[13]) diets has been associated with better cognitive outcomes and reduced dementia risk, whereas diets high in ultra-processed foods, saturated fats, and added sugars may be detrimental (Wieckowska-Gacek et al., 2021[127]). Nonetheless, findings across studies are heterogeneous, and uncertainty remains regarding optimal dietary components, critical exposure windows, and the extent to which observed associations reflect causal relationships.

Similarly, evidence linking other lifestyle factors to cognitive outcomes has expanded rapidly. Regular physical activity and sustained cognitive and social engagement are generally associated with reduced risk of cognitive decline, while smoking, excessive alcohol consumption, physical inactivity, poor sleep, social isolation, and loneliness have been linked to increased risk (Dominguez et al., 2021[22]; Livingston et al., 2024[68]; Jones et al., 2024[53]). However, interpretation of these associations is complicated by methodological challenges, including reverse causation, competing risks, residual confounding, and variability in exposure definitions and outcome ascertainment. Moreover, evidence quality varies considerably across lifestyle domains, with some factors supported by robust meta-analytic data and others by more limited or inconsistent findings.

In recent years, the publication of large prospective cohort studies, updated meta-analyses, umbrella reviews, and multidomain intervention trials has substantially advanced the field. These studies have refined estimates of association, explored dose-response relationships, and provided insights into potential biological mechanisms and interactions among lifestyle factors. At the same time, they have highlighted important gaps, including limited evidence from randomized controlled trials (RCTs), underrepresentation of diverse populations, and insufficient consideration of life-course timing and cumulative exposure.

Given the rapid evolution of the evidence base, an updated synthesis is warranted. This narrative review aims to critically summarize current evidence on lifestyle factors and dietary patterns associated with cognitive decline and dementia risk, with a focus on recent high-quality observational studies and evidence syntheses. By integrating recent findings across domains, this review seeks to clarify areas of consensus and uncertainty, inform prevention strategies, and identify priorities for future research and public health action.

## 2. Lifestyle-Related Determinants

**Figure 2:**
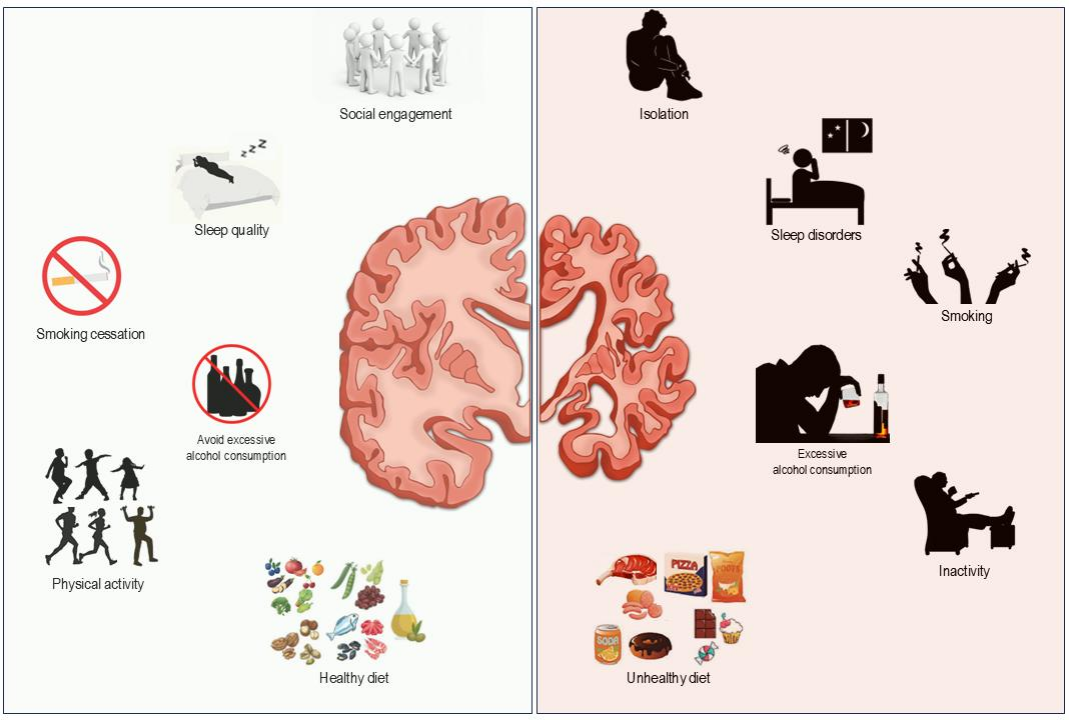
On the left are lifestyle and dietary factors currently considered protective against cognitive decline and dementia, while on the right are the corresponding adverse factors that contribute to disease development.

Lifestyle factors are central to dementia prevention (Figure 2(Fig. 2)). Evidence suggests that physical activity, healthy diet, smoking cessation, avoiding heavy alcohol consumption, adequate sleep, and social and cognitive engagement are associated with better cognitive outcomes and lower dementia risk, likely through effects on vascular, inflammatory, metabolic, and neuroplastic pathways. Despite limited causal evidence from long-term trials, consistent observational findings support a multidomain lifestyle approach to reducing dementia risk at the population level.

### 2.1 Social engagement/isolation

Social isolation or infrequent social contact has been identified as a risk factor for dementia. Two previous systematic reviews from 2015 and 2018 found that less frequent social contact was associated with increased dementia risk, with reported relative risks ranging from 1.18 to 1.57 (Penninkilampi et al., 2018[88]; Kuiper et al., 2015[58]). Differences in follow-up duration may explain variability, as several studies included in these meta-analyses had short follow-up periods, increasing the likelihood of reverse causation. However, two later UK Biobank studies with longer follow-up (8.8 and 12 years) showed higher dementia risk among individuals who were more socially isolated at baseline, defined by living alone, infrequent contact with family or friends, and lack of weekly group activities (Elovainio et al., 2022[24]; Shen et al., 2022[103]). Former intervention trials using facilitator-led group activities to increase social contact have shown mixed effects on cognition. A Finnish RCT in lonely adults aged 75 years or older found a small cognitive improvement (Pitkala et al., 2011[89]), whereas studies from the USA (Park et al., 2014[86]) and China (Mortimer et al., 2012[79]) found no cognitive benefit.

As shown in Table 1(Tab. 1) (References in Table 1: Cunha et al., 2024[17]; Jones et al., 2024[53]; Joshi et al., 2024[54]; Lazzari and Rabottini, 2022[59]; Lucchetti et al., 2024[72]; Wang et al., 2023[122]), more recent meta-analytic evidence indicates a consistent association between social factors and cognitive outcomes across diverse populations and study designs (Luchetti et al., 2024[72]; Cunha et al., 2024[17]; Joshi et al., 2024[54]; Jones et al., 2024[53]; Wang et al., 2023[122]; Lazzari and Rabottini, 2022[59]). Overall, higher levels of social engagement-including formal participation, frequent social contact, and broader social networks-were associated with a reduced risk of cognitive decline and dementia, whereas loneliness and social isolation are linked to an increased risk. Effect sizes are generally modest to moderate but consistent in direction, with stronger associations observed for loneliness and social isolation in relation to all-cause dementia and dementia subtypes, including Alzheimer's disease and vascular dementia.

Notably, large pooled analyses (e.g., Luchetti et al., 2024[72]; Wang et al., 2023[122]) demonstrated that associations persist after adjustment for key confounders such as depression, suggesting an independent contribution of social factors to cognitive health. However, substantial heterogeneity, variability in exposure definitions, and predominantly observational designs limit causal inference. Evidence quality also varies by exposure, with formal social participation showing potential cognitive benefits but supported by low-certainty evidence. Collectively, these findings support social engagement and isolation as potentially modifiable factors in dementia risk, while underscoring the need for more rigorous longitudinal and interventional studies to clarify mechanisms and causality.

### 2.2 Sleep-related disturbances

Sleep disturbance is another potentially modifiable risk factor for dementia. Growing evidence links sleep disorders to cognitive decline (Bubu et al., 2017[10]; Shi et al., 2018[105]; Wu et al., 2019[130]), highlighting sleep's role in brain waste clearance and neuroplasticity, and circadian regulation (Parhizkar et al., 2023[85]; Brown et al., 2016[9]). Sleep deprivation and circadian disruption impair clearance of β-amyloid and tau, increase oxidative stress, and reduce melatonin, promoting neurodegeneration and increasing dementia risk (Mian et al., 2025[77]). Identifying sleep-related risk factors may therefore support targeted dementia prevention.

Longitudinal studies link sleep disturbances to higher risk of all-cause dementia, Alzheimer's disease, and vascular dementia (Shi et al., 2018[105]), with specific disorders showing distinct associations (e.g., insomnia with Alzheimer's disease and sleep-disordered breathing with multiple dementia types). Observational data suggest that treating obstructive sleep apnea may reduce dementia risk (Dunietz et al., 2021[23]), but clinical trials have not shown that continuous positive airway pressure reverses Alzheimer's disease-related biomarkers (Mullins et al., 2020[80]).

Two previous meta-analyses, using varied definitions of short (≤ 7 h) and long (> 8 h) sleep and follow-up periods under 10 years, reported an inverted U-shaped association between sleep duration and dementia risk, though these findings may be influenced by reverse causation (Liang et al., 2019[65]; Fan et al., 2019)[27].

Beyond sleep duration, sleep quality-particularly sleep apnea-has been linked to dementia risk. A meta-analysis of 11 studies (over 1.3 million participants) found a higher dementia risk in people with sleep apnea, though few studies adjusted for obesity (Guay-Gagnon et al., 2022[37]). These findings suggest potential value in screening for dementia among individuals with sleep apnea.

Across recent meta-analyses (Table 2(Tab. 2); References in Table 2: Howard et al., 2024[40]; Jones et al., 2024[53]; Khaing et al., 2025[55]; Ungvari et al., 2025[119]; Yang et al., 2024[131]; Zhang et al., 2025[133]), sleep alterations emerge as consistent and clinically relevant correlates of cognitive decline and dementia, with converging evidence indicating that both qualitative sleep disorders (e.g., insomnia, obstructive sleep apnea) and quantitative sleep disturbances (e.g., short or long sleep duration, excessive daytime sleepiness) are associated with increased risk of adverse cognitive outcomes. The largest meta-analysis to date by Ungvari et al. (2025[119]) demonstrates that sleep disorders as a broad category confer a substantially elevated risk of all-cause dementia and its major subtypes, with pooled hazard ratios exceeding 1.5. Notably, both obstructive sleep apnea and insomnia show robust associations with Alzheimer's disease and vascular dementia, supporting the hypothesis that disrupted sleep physiology may contribute to both neurodegenerative and cerebrovascular pathways of cognitive impairment.

Complementing these findings, Zhang et al. (2025[133]) extend the evidence base by demonstrating that multiple dimensions of sleep disturbance-including abnormal sleep duration, poor sleep quality, excessive daytime sleepiness, and sleep-related movement disorders-are independently associated with cognitive decline and dementia outcomes. The particularly strong association observed for sleep-related movement disorders and vascular dementia suggests a potential role for shared vascular or neuroinflammatory mechanisms. Importantly, this work underscores that dementia risk is not confined to sleep duration alone, but reflects a broader spectrum of sleep dysfunction.

Meta-analyses focusing on excessive daytime sleepiness and long sleep duration, such as Khaing et al. (2025[55]), further highlight that excessive sleep propensity is associated with increased risks of both cognitive decline and all-cause dementia. However, the attenuation of associations after adjustment and the wide confidence intervals for long sleep duration suggest potential reverse causality or prodromal effects, rather than a purely causal role. This interpretation is reinforced by Howard et al. (2024[40]), who report stronger associations between short sleep and dementia in studies with shorter follow-up periods, implying that sleep changes may reflect early neurodegenerative processes rather than antecedent risk factors.

Finally, dose-response analyses by Yang et al. (2024[131]) provide compelling evidence of a non-linear (U-shaped) relationship between sleep duration and cognitive decline, with both short and long sleep durations, as well as trajectories toward prolonged sleep, conferring elevated risk. These findings align with emerging models in which sleep alterations may act both as modifiable risk factors and as early markers of neurodegeneration, depending on their timing, persistence, and underlying etiology.

Collectively, these recent meta-analyses support the inclusion of sleep disturbances among key factors linked to dementia risk while highlighting substantial heterogeneity, potential reverse causation, and the need for long-term prospective studies and intervention trials to clarify causality. Differentiating between sleep disturbances as prodromal symptoms versus modifiable targets for prevention remains a critical priority for future research.

### 2.3 Smoking habits

Smoking is a well-established modifiable risk factor for a wide range of chronic diseases and has increasingly been implicated in cognitive decline and dementia. In the three Lancet Commission reports (Livingston et al., 2024[68]; Livingston et al., 2020[69]; Livingston et al., 2017[70]), smoking is considered a significant risk factor for cognitive decline and dementia. Tobacco smoke contains numerous neurotoxic and pro-inflammatory compounds that contribute to oxidative stress, vascular damage, and neuroinflammation-pathways strongly linked to neurodegeneration (Ewees et al., 2025[26]; Mazzone et al., 2010[74]; Toda and Okamura, 2016[115]). Epidemiological studies consistently show that current smoking is associated with an increased risk of all-cause dementia, Alzheimer's disease, and vascular dementia, with evidence of a dose-response relationship, as discussed below. Smoking may also accelerate cognitive aging by promoting cerebrovascular disease, atherosclerosis, and impaired cerebral perfusion (Ewees et al., 2025[26]). Importantly, smoking cessation appears to attenuate dementia risk over time, with former smokers demonstrating lower risk compared with current smokers (Bloomberg et al., 2025[7]; Jeong et al., 2023[50]; Lee et al., 2022[60]; Cheon et al., 2022[15]), underscoring the potential benefits of smoking prevention and cessation for brain health across the life course.

Recent quantitative summary evidence examining smoking and dementia risk remains limited. No quality meta-analyses published in recent years have examined smoking as a standalone exposure. Instead, most recent quantitative syntheses tend to combine smoking with other substance-use exposures or broader categories of risk factors for cognitive disorders (Jones et al., 2024[53]), or they appear within narrative or systematic reviews rather than as stand-alone meta-analyses.

Earlier meta-analyses, most notably the 2015 study by Zhong et al. (2015[137]), therefore remain relevant in contemporary literature but do not include recent studies that we will review below. Nevertheless, these former studies provide a robust quantitative estimate of the association between smoking and risk of all-cause dementia as well as dementia subtypes, including Alzheimer's disease and vascular dementia. Importantly, findings from more recent narrative reviews and large original studies (Table 3(Tab. 3); References in Table 3: Bloomberg et al., 2025[7]; Chen et al., 2023[12]; Cheon et al., 2022[15]; Jeong et al., 2023[50]; Lee et al., 2022[60]; Li et al., 2025[64]; Lin et al., 2025[66]; Meysami et al., 2025[76]; Myrstad et al., 2025[81]; Raggi et al., 2022[91]; Zhang et al., 2021[134]), including the study published in 2025 by Meysami et al. (2025[76]), consistently reaffirm that smoking is associated with an increased risk of dementia. Reported effect sizes in these newer studies are generally comparable to those observed in earlier meta-analytic work, with some evidence suggesting modification of risk by genetic factors such as APOE (apolipoprotein E) ε4 status.

Table 3(Tab. 3) comprehensively summarizes recent cohort studies linking smoking with cognitive decline and dementia risk (Zhang et al., 2021[134]; Cheon et al., 2022[15]; Lee et al., 2022[60]; Raggi et al., 2022[91]; Chen et al., 2023[12]; Jeong et al., 2023[50]; Bloomberg et al., 2025[7]; Li et al., 2025[64]; Lin et al., 2025[66]; Meysami et al., 2025[76]; Myrstad et al., 2025[81]). Across diverse populations, study designs, and follow-up durations, current smoking consistently emerges as a significant risk factor for cognitive impairment, accelerated cognitive decline, and incident dementia, with effect sizes typically ranging from ~30-80 % increased risk (HRs/RRs 1.31-1.78). Early-life and midlife smoking (Lin et al., 2025[66]), higher cumulative exposure (pack-years) (Raggi et al., 2022[91]), and persistent smoking are particularly associated with greater risk, while smoking cessation-especially sustained quitting-(Cheon et al., 2022[15]; Lee et al., 2022[60]; Jeong et al., 2023[50]; Bloomberg et al., 2025[7]) is linked to reduced cognitive decline and lower dementia risk, though former smokers may still retain some residual risk. Structural brain studies support a mechanistic basis, showing lower gray and white matter volumes in smokers, partly mediated by body mass index (BMI) (Meysami et al., 2025[76]). Genetic factors, such as APOE-ε4 status and polygenic risk, modulate the smoking-dementia relationship, with smoking effects more pronounced in non-ε4 carriers (Zhang et al., 2021[134]). Furthermore, smoking partially mediates the socioeconomic gradient in dementia risk (Raggi et al., 2022[91]), highlighting its role as a modifiable risk factor with both public health and clinical implications. Collectively, these findings underscore the importance of early prevention, targeted interventions, and smoking cessation programs to reduce cognitive decline and dementia burden across the lifespan.

### 2.4 Excessive alcohol consumption

The evidence on alcohol use and subsequent dementia risk is mixed and remains subject to ongoing debate. Many observational studies suggest that light to moderate alcohol consumption is associated with a lower risk of dementia compared with abstinence (Ilomaki et al., 2015[45]; Mewton et al., 2023[75]), although this apparent protective effect may partly reflect residual confounding, selection bias, or misclassification of former drinkers as abstainers. In contrast, several studies indicate that heavy alcohol consumption and alcohol use disorders are associated with an increased risk of dementia, including both Alzheimer's disease and vascular dementia, likely through mechanisms involving neurotoxicity, nutritional deficiencies, liver dysfunction, and cerebrovascular damage (Schwarzinger et al., 2018[100]; Rehm et al., 2019[93]). However, not all studies have observed this association (Ilomaki et al., 2015[45]), and inconsistencies across findings may be attributable to differences in study design, exposure assessment, drinking patterns, follow-up duration, and population characteristics. As a result, the relationship between alcohol consumption and dementia remains complex, with uncertainty regarding thresholds of harm, potential dose-response effects, and the extent to which observed associations reflect causal relationships.

In the 2020 report from the Lancet Commission on Dementia Prevention, Intervention, and Care, midlife alcohol consumption exceeding 21 UK units per week was associated with a significant 18 % increased risk of dementia compared with lighter drinking (Livingston et al., 2020[69]), similar to that found by the same Commission in 2024 (RR 1.2, 95 % CI 1.0-1.5) (Livingston et al., 2024[68]). This finding was supported by a meta-analysis of over 131,000 participants from four European countries, which reported a significant 22 % higher dementia risk among heavier drinkers (> 21 units/week) compared with lighter drinkers (Kivimaki et al., 2020[56]). Consistently, evaluation of 28 systematic reviews concluded that heavy alcohol use was associated with increased all-cause dementia risk and reduced gray matter volume (Rehm et al., 2019[93]).

Some studies report similar dementia risk in heavy drinkers and non-drinkers, though many non-drinkers are former heavy drinkers (John et al., 2021[52]). A large Japanese cohort found that both abstinence and heavy midlife drinking were associated with higher dementia risk compared with light drinking (Shimizu et al., 2023[106]). Meta-analytic evidence indicates lower dementia risk among occasional to moderate drinkers, but not among heavy drinkers, relative to non-drinkers (Mewton et al., 2023[75]). Mendelian randomization suggests that alcohol consumption may causally influence earlier Alzheimer's disease onset and that associations with abstinence likely reflect survivor or reverse-causation bias (Andrews et al., 2020[1]), helping to explain the commonly observed J-shaped relationship.

A large South Korean cohort found that sustained heavy drinking increased dementia risk, while reducing intake lowered risk; mild or moderate drinking was associated with lower risk than sustained non-drinking (Jeon et al., 2023[49]), again, possibly reflecting former heavy drinkers among abstainers. Overall, reducing excessive alcohol consumption or maintaining light drinking was associated with lower dementia risk than continued heavy drinking, and there is no clear evidence that abstinence itself increases dementia risk; observed excess risk among non-drinkers likely reflects misclassification or reverse causation.

In recent years, new evidence has emerged from large-scale meta-analyses, cohort studies, and genetic investigations examining alcohol consumption and cognitive outcomes, as synthesized in Table 4(Tab. 4) (References in Table 4: Huang et al., 2025[42]; Jones et al., 2024[53]; Topiwala et al., 2026[116]; Zarezadeh et al., 2024[132]; Zhang et al., 2025[135]). Confirming previous evidence, observational meta-analyses consistently suggest a J-shaped association, with light-to-moderate alcohol intake associated with a lower risk of all-cause dementia and Alzheimer's disease, and heavy or excessive consumption associated with increased risk across dementia subtypes. However, findings from genetically informed analyses, particularly Mendelian randomization (Topiwala et al., 2026[116]), challenge the apparent protective effect of low-level drinking by demonstrating a dose-dependent, likely causal increase in dementia risk with higher alcohol intake. Stratified analyses further indicate effect modification by APOE ε4 status (Huang et al., 2025[42]), with stronger harmful associations of heavy drinking among carriers and protective observational associations of light-to-moderate intake confined to noncarriers. Taken together, these results highlight substantial heterogeneity by drinking level and genetic susceptibility, and underscore the likelihood that observational protective effects may reflect residual confounding or reverse causation rather than true neuroprotection. Nonetheless, there is broad consensus that excessive alcohol consumption is clearly associated with an increased risk of dementia.

### 2.5 Physical activity/inactivity

Exercise across the life course appears to support cognitive health, potentially through improved cerebral blood flow, reduced hypertension, increased nitric oxide availability, enhanced brain plasticity, and lower neuroinflammation (Huuha et al., 2022[44]). Beyond possible cognitive benefits, physical exercise in older adults is linked to better balance, fewer falls (Sherrington et al., 2020[104]), improved mood (Singh et al., 2023[109]), reduced mortality (Fukushima et al., 2024[33]), and enhanced functional capacity (Niyazi et al., 2024[83]). There is evidence that greater engagement in moderate-to-vigorous activity has also been associated with larger brain volumes compared with lower or no activity (Raji et al., 2024[92]). Nevertheless, assessing the protective role of physical activity against cognitive decline and dementia is methodologically challenging. Reverse causation may inflate observed associations, as early cognitive decline can lead to reduced activity. Measurement error is common because physical activity is often self-reported and varies in type, intensity, and duration. Residual confounding is also substantial, as physically active individuals differ from inactive individuals in socioeconomic, lifestyle, and health factors that influence dementia risk, and competing risks such as premature mortality further complicate interpretation. Evidence is additionally limited by heterogeneity across studies in exposure assessment, follow-up, and outcome definitions, as well as uncertainty about the most relevant life-course timing of activity. Available RCTs are few, typically short, and focus on cognitive outcomes rather than incident dementia. Finally, biological heterogeneity across dementia subtypes may lead to differential effects of physical activity, contributing to ongoing uncertainty about causality. For example, physical activity in midlife has not consistently been linked to cognitive performance, whereas associations are more often observed in later life (Sabia et al., 2017[97]; Greendale et al., 2021[36]); however, these findings may partly reflect reverse causation. A longitudinal study of 1,718 women followed for a median of 11.9 years found that higher physical activity was associated with less cognitive decline, although this association was attenuated after adjustment for diabetes and hypertension (Greendale et al., 2021[36]).

Former meta-analyses of observational studies of exercise indicated benefit of exercise on prevention of Alzheimer's disease and dementia (Hersi et al., 2017[39]; Zotcheva et al., 2018[138]; Sofi et al., 2011[110]; Hamer and Chida, 2009[38]), and several more recent studies confirm this benefit. For example, a large meta-analysis of 58 studies involving over 250,000 participants found that physical activity was associated with lower risks of all-cause dementia and Alzheimer's disease, with evidence extending to long follow-up periods and across ages. Risk reductions were greatest when moving from sedentary behavior to some activity, with some evidence for vascular dementia mainly in shorter follow-up periods (Iso-Markku et al., 2022[46]). Cohort studies further suggest that sustained or increased physical activity across adulthood is associated with better late-life cognition and lower dementia risk (James et al., 2023[48]; Tari et al., 2022[113]), although some associations attenuate after adjustment for cardiometabolic conditions (Greendale et al., 2021[36]). A 5-year RCT of 945 older adults found no overall differences in cognition or mild cognitive impairment (MCI) risk between control, moderate-intensity, and high-intensity exercise groups, although men in the exercise groups had a lower risk of MCI and slightly better cognitive scores than controls (Zotcheva et al., 2018[138]). Declines in cardiorespiratory fitness were associated with higher odds of MCI (Zotcheva et al., 2018[138]). Overall, evidence from RCTs suggests only small cognitive benefits of exercise, with effects potentially dependent on activity type and intensity, supporting population-level strategies to reduce physical inactivity (Ciria et al., 2023[16]).

Table 5(Tab. 5) (References in Table 5: Feter et al., 2023[29]; Iso-Markku et al., 2022[46]; Jiang et al., 2025[51]; Jones et al., 2024[53]; Luo et al., 2025[73]; Reparaz-Escudero et al., 2024[94]; Zhang et al., 2023[136]) provides a comprehensive overview of recent meta-analyses examining the role of physical activity and sedentary behavior in cognitive decline and dementia risk. Across multiple large-scale cohort studies and systematic reviews, higher levels of physical activity-particularly moderate-to-vigorous or high-intensity activity-consistently reduced the risk of Alzheimer's disease, all-cause dementia, and vascular dementia, with risk reductions ranging from 14 % to 44 % depending on intensity (Jiang et al., 2025[51]; Reparaz-Escudero et al., 2024[94]; Iso-Markku et al., 2022[46]; Zhang et al., 2023[136]). Dose-response analyses further support a linear benefit, with each 10 MET-h/week increment associated with roughly 15 % lower Alzheimer's disease risk (Jiang et al., 2025[51]). Evidence from long-term follow-up studies suggests that physical activity remains protective even after accounting for reverse causation, highlighting its potential as a low-cost, modifiable preventive strategy.

Conversely, physical inactivity and sedentary behavior were consistently associated with increased dementia risk. Population attributable fraction estimates suggest that 6-17 % (Feter et al., 2023[29]) of dementia cases could be linked to inactivity, emphasizing its public health relevance. While exercise-only interventions produced minimal cognitive benefits in RCTs, multidomain interventions combining exercise with other lifestyle components yielded small but positive effects, suggesting that physical activity may be most effective as part of comprehensive preventive strategies.

Overall, these findings reinforce physical activity as a key modifiable factor for dementia prevention, with both observational and interventional evidence supporting its protective role, while sedentary behavior emerges as an independent risk factor.

## 3. Dietary Patterns

**Figure 3:**
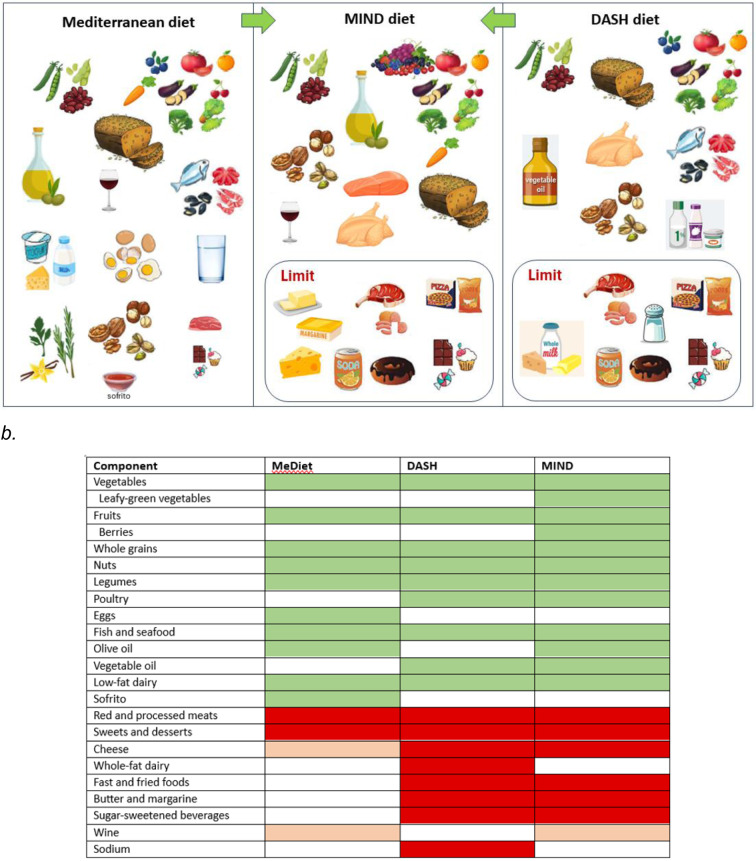
(a) Components of the Mediterranean (left), MIND (center), and DASH (right) diets are shown. Several elements overlap, reflecting the fact that the MIND diet is derived from both the Mediterranean and DASH dietary patterns. DASH indicates Dietary Approaches to Stop Hypertension; MIND, Mediterranean-DASH Intervention for Neurodegenerative Delay. (b) Overlap of some components of the three dietary patterns. Green indicates foods to be eaten frequently, red indicates foods to be eaten infrequently, and pink indicates foods that should be limited.

Observational evidence suggests that the MeDiet, the DASH diet, and their hybrid, the MIND diet (components are illustrated in Figure 3(Fig. 3)), may confer neuroprotective benefits (Chen et al., 2023[13]; Seabrook et al., 2025[101]; Seago et al., 2025[102]; Dominguez et al., 2021[22]), though findings are inconsistent across populations with varying baseline cognitive status. For example, one study reported that overall adherence to the DASH diet was not associated with cognitive performance or cognitive change (Daniel et al., 2021[18]), and a more recent trial observed no differences in cognitive trajectories between participants following the MIND diet and those on a control diet, possibly due to the relatively short follow-up period of three years (Barnes et al., 2023[5]). This variability highlights the complex relationship between diet and brain health. As with exercise, assessing diet's protective effects on cognitive decline and dementia is challenging due to methodological and biological factors. Dietary intake is often self-reported and variable over time, leading to measurement error. People consume complex food combinations, making it difficult to isolate the effects of individual nutrients or their blends, while residual confounding from lifestyle, socioeconomic, and health factors may bias results. Long dementia latency and short-term dietary assessments, along with reverse causation from preclinical cognitive changes affecting diet, further complicate interpretation.

In fact, the World Health Organization's 2019 conditional recommendation for the MeDiet for dementia prevention reflects ongoing uncertainty in the evidence (World Health Organization, 2019[129]), which also likely explains why diet was classified as a potential but insufficiently supported risk factor and excluded from the life-course population attributable fraction estimates in the 2024 Lancet Commission on dementia prevention report (Livingston et al., 2024[68]). Nevertheless, literature on this topic continues to grow in recent years. A systematic review and meta-analysis of 16 cohort studies (follow-up 2.2-41 years) found that higher overall diet quality was associated with an 18 % reduced dementia risk (Liu et al., 2020[67]), with similar findings in studies with longer follow-up and for Alzheimer's disease. However, continuous MeDiet scores showed no significant association (Liu et al., 2020[67]). A larger meta-analysis of three cohorts with over 200,000 participants reported that greater adherence to the MIND diet was linked to a 17 % lower dementia risk (Chen et al., 2023[13]). Another review found protective effects of the MeDiet on global cognitive decline in nearly half of studies, with more limited evidence for incident dementia and Alzheimer's disease (Townsend et al., 2023[118]).

With the aim of summarizing the findings of the vast literature on this topic, we have reviewed evidence from recent systematic reviews and meta-analyses (Table 6(Tab. 6); References in Table 6: Antonelli and Donelli, 2022[3]; Chen et al., 2023[13]; Fekete et al., 2025[28]; Fu et al., 2022[32]; Nucci et al., 2024[84]; Wang et al., 2025[124]; Zuliani et al., 2026[139]). As shown, the recent meta-analytic evidence indicates that adherence to healthy dietary patterns is associated with a reduced risk of cognitive decline and dementia, with notable differences across diet types. The MeDiet, for which there is a greater body of literature, shows robust and reproducible associations with lower risks of Alzheimer's disease, MCI, and all-cause dementia, with relative risk reductions generally ranging from 7 % to 31 % (Zuliani et al., 2026[139]; Fekete et al., 2025[28]; Nucci et al., 2024[84]; Fu et al., 2022[32]; Antonelli and Donelli, 2022[3]). These associations are supported by both observational cohorts and umbrella reviews, although evidence from RCTs remains limited and primarily confined to modest improvements in specific cognitive domains.

By contrast, evidence for the DASH diet-currently lacking a meta-analysis-is more inconsistent, with no clear overall association with dementia risk among older adults. The MIND diet, which integrates elements of the Mediterranean and DASH diets, demonstrates the most consistent protective associations in the few meta-analyses available, including a 20-40 % reduction in dementia and Alzheimer's disease risk. Notably, the protective effect of the MeDiet appears stronger in studies with shorter follow-up durations or specific diagnostic criteria, suggesting potential influences of study design, reverse causation, and outcome ascertainment.

Overall, these findings support adherence to Mediterranean-based dietary patterns-particularly the MIND diet-as promising non-pharmacological strategies for dementia prevention. However, heterogeneity across studies and the relative scarcity of long-term RCTs highlight the need for further high-quality, longitudinal and interventional research to strengthen causal inference and inform dietary recommendations.

Other dietary patterns with some evidence of neuroprotection include plant-based diets, the Nordic diet, and anti-inflammatory diets (Bigras et al., 2025[6]; de Crom et al., 2023[19]; Townsend et al., 2024[117]; Chen et al., 2019[14]). Predominantly plant-based diets have been linked to favorable cognitive outcomes. A recent systematic review and meta-analysis reported that greater adherence to healthful plant-based diets was associated with lower odds of cognitive impairment and a modest reduction in dementia risk, although results varied across studies. Higher-quality plant-based diet scores, reflecting greater intake of healthy plant foods, showed stronger protective associations than overall plant-based indices (Bigras et al., 2025[6]). However, evidence is not uniform, as some population-based studies found no clear association between general plant-based eating patterns and dementia risk, suggesting that the quality of plant foods may be more influential than plant-based adherence alone (de Crom et al., 2023[19]).

The traditional Nordic diet, characterized by root vegetables, whole grains (rye, barley, oats), berries, fish, and rapeseed oil, has been linked to slower cognitive decline and longer dementia free survival in older adults in some Scandinavian cohorts. This pattern shares key features with other healthy diets (e.g., high in plant foods and unsaturated fats), suggesting that beneficial effects on cognition may generalize beyond Mediterranean style eating (Townsend et al., 2024[117]).

Other healthy dietary patterns, such as anti-inflammatory or prudent diets rich in fruits, vegetables, lean proteins, and whole foods, have been linked to better cognitive outcomes in some studies, though they largely overlap with MeDiet and plant-based diets (Chen et al., 2019[14]).

## 4. Lifestyle-Related Diseases and Risk Factors

**Figure 4:**
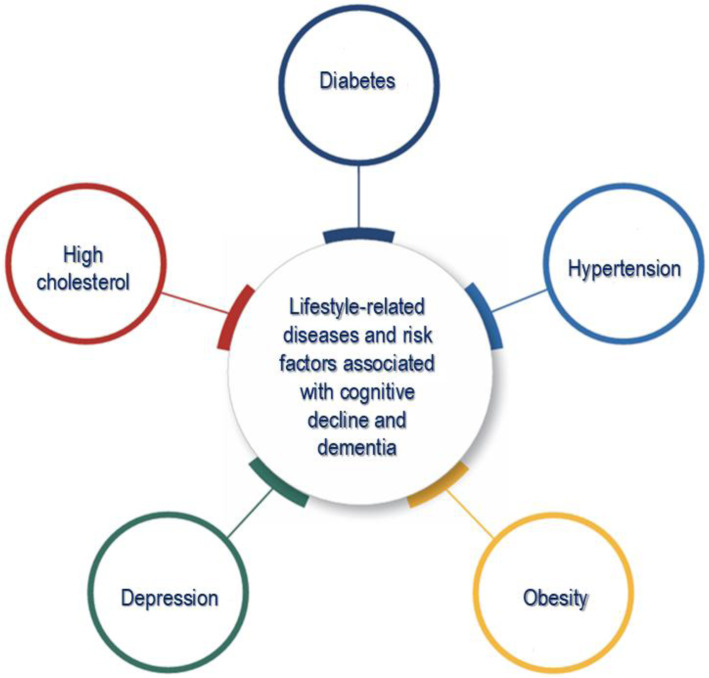
Some of the most common lifestyle-related diseases and risk factors linked to cognitive decline and dementia are shown, highlighting conditions and behaviors that influence brain health across the life course and contribute to the development and progression of neurodegenerative disease.

A growing body of epidemiological and mechanistic evidence indicates that lifestyle- and nutrition-related modifiable cardiometabolic and psychosocial conditions (Figure 4(Fig. 4)) significantly contribute to the development and progression of cognitive decline and dementia (Livingston et al., 2024[68]). These associations are robust across populations and represent key targets for primary prevention.

Large-scale RCTs have shown that lifestyle modification programs significantly lower diabetes risk in individuals at high-risk (Lee et al., 2025[61]). Type 2 diabetes is consistently associated with an elevated risk of dementia, particularly vascular dementia and all-cause dementia, likely due to chronic hyperglycemia, insulin resistance, and vascular injury (de la Monte et al., 2018[20]; Cao et al., 2024[11]; Michailidis et al., 2022[78]). Diabetes promotes cerebrovascular disease, oxidative stress, and inflammation, which accelerate neurodegeneration and cognitive impairment (Michailidis et al., 2022[78]; de la Monte et al., 2018[20]). Epidemiological studies show that individuals with diabetes have a notably higher risk of progressing from MCI to dementia compared with non-diabetics (Ding et al., 2024[21]). Diabetes may contribute to dementia through several mechanisms: insulin resistance, where disrupted brain insulin signaling affects amyloid β metabolism; vascular damage, increasing stroke risk and microvascular injury that lowers cerebral perfusion; and chronic inflammation, which can accelerate neuronal loss (de la Monte et al., 2018[20]; Michailidis et al., 2022[78]). However, Mendelian randomization data suggest that diabetes per se may not directly increase Alzheimer's disease risk independent of metabolic intermediates like insulin and cholesterol, highlighting complexity in causal pathways (Wang et al., 2024[123]). A recent meta-analysis of 15 studies (10.1 million participants; 8.8 million with diabetes) showed that diabetes was associated with a 59 % significantly increased risk of dementia. Diabetes duration < 5 years (RR 1.29, 95 % CI 1.20-1.39) and hypoglycemia (RR 1.56, 95 % CI 1.13-2.16) were associated with higher dementia risk, while glycemic control measures showed no significant effect (Wang et al., 2024[123]).

High blood pressure, especially during midlife, is one of the most consistent vascular risk factors linked to later cognitive decline and dementia (Huang and Aronow, 2024[41]; Levine et al., 2020[63]). Elevated systolic blood pressure contributes to small vessel disease, white matter hyperintensities, and microinfarcts, which are neuropathological substrates of both vascular dementia and Alzheimer's disease (Ungvari et al., 2021[120]). Furthermore, blood pressure lowering with antihypertensive therapy, compared to control, was significantly linked to a reduced risk of dementia or cognitive impairment in a meta-analysis of 14 RCTs (Hughes et al., 2020[43]).

Obesity, particularly in midlife, is associated with higher dementia risk (Qu et al., 2020[90]; Emmerzaal et al., 2015[25]). Adiposity contributes to systemic inflammation, dysregulated adipokine profiles, insulin resistance, and cerebrovascular disease, all of which can adversely affect brain structure and function (Patel and Edison, 2024[87]).

High total cholesterol and dyslipidemia in midlife have been linked to increased dementia risk, particularly Alzheimer's disease (Wee et al., 2023[125]; Iwagami et al., 2021[47]). Proposed mechanisms include cholesterol-mediated effects on cell membrane composition and amyloid processing (Gamba et al., 2015[34]), as well as dyslipidemia-related cerebrovascular atherosclerosis, which may reduce cerebral blood flow (Siegler et al., 2025[107]). Although observational data support an association, clinical evidence on the cognitive benefits of lipid-lowering therapies remains mixed and requires further investigation. Meta-analyses reported inconsistent evidence suggesting that elevated LDL cholesterol in midlife-but not in late life-may be associated with an increased risk of cognitive decline, all-cause dementia, and Alzheimer's disease (Anstey et al., 2017[2]; Wood et al., 2014[128]).

Depression is increasingly recognized as both a risk factor and early symptom of cognitive decline. Longitudinal cohort studies have found that depressive symptoms are associated with an elevated risk of subsequent dementia, independent of other health conditions (Stafford et al., 2022[112]). Potential mechanisms include chronic stress and dysregulation of hypothalamic-pituitary-adrenal axis leading to hippocampal damage, inflammatory pathways that overlap with neurodegenerative processes, and reduced cognitive reserve due to behavioral and lifestyle changes associated with depression (Borges de Souza et al., 2025[8]). Depression may interact synergistically with metabolic risk factors to further elevate dementia risk, underscoring the importance of integrated care. Depression is closely intertwined with lifestyle factors. Unhealthy behaviors-such as poor diet quality, physical inactivity, inadequate sleep, smoking, excessive alcohol consumption, and chronic stress-are associated with an increased risk of developing depressive symptoms, whereas healthier patterns, including regular physical activity, balanced nutrition, and good sleep hygiene, appear to be protective. Although depression has important biological and genetic underpinnings, lifestyle plays a substantial modulatory role in its onset, severity, and persistence. Moreover, lifestyle and depression often interact bidirectionally, creating a self-reinforcing cycle in which depressive symptoms lead to deteriorating health behaviors, which in turn further exacerbate depression and impede recovery (Sato et al., 2025[99]). In essence, while not the only cause, lifestyle factors are powerful contributors and modulators of depression, acting as significant risk or protective elements.

## 5. Perspective

**Figure 5:**
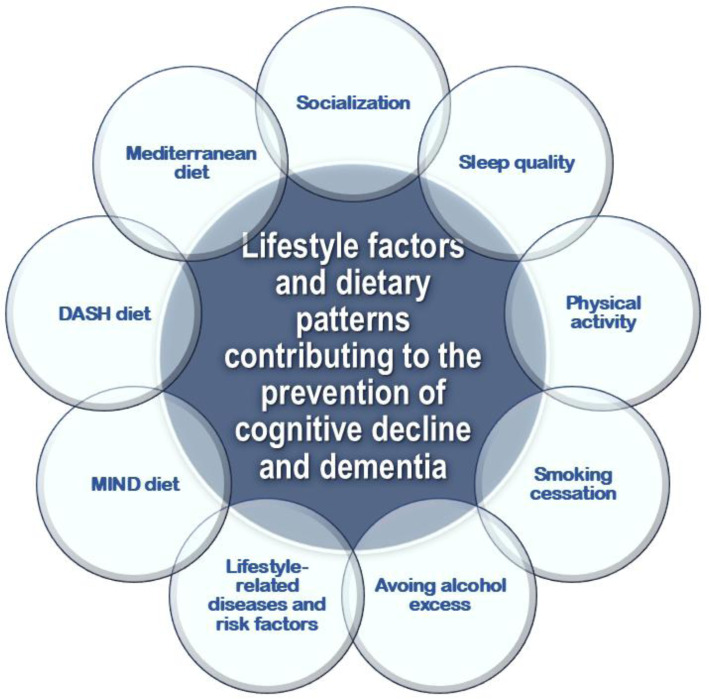
Lifestyle factors discussed in this article that contribute to the prevention of cognitive decline and dementia are summarized, emphasizing their combined and potentially synergistic roles and underscoring the importance of considering them within multimodal prevention and intervention strategies.

Lifestyle factors are central to the prevention of cognitive decline and dementia. Because complex diseases develop through the cumulative interaction of multiple influences over time, effective prevention is likely to require integrated lifestyle strategies, such as those reviewed in this article (Figure 5(Fig. 5)), many of which show evidence of potential neuroprotective effects.

Successive updates of the Lancet Commission on Dementia Prevention, Intervention, and Care have progressively broadened understanding of modifiable, largely lifestyle-related dementia risk factors across the life course. Emerging evidence has expanded the framework beyond individual medical conditions to include biological, sensory, environmental, and lifestyle factors. The number of identified modifiable risks has increased from 9 in the first report (Livingston et al., 2017[70]) to 14 in the most recent update (Livingston et al., 2024[68]), reflecting growing recognition of multiple, interacting pathways to dementia, including vascular disease, sensory impairment, environmental exposures, and health-related behaviors. Correspondingly, the estimated proportion of preventable dementia cases has risen from ~35 % to 40 %, and most recently to 45 % (Livingston et al., 2024[68]; Livingston et al., 2020[69]; Livingston et al., 2017[70]), underscoring the potential impact of comprehensive, lifespan-wide risk reduction strategies.

Evidence supporting multidomain interventions as a non-pharmacological strategy for dementia prevention is rapidly growing (Sakurai et al., 2025[98]). However, a definitive answer to whether dementia can be prevented remains elusive. To date, most studies have relied on changes in cognitive function as surrogate outcomes rather than dementia incidence, which would require longer follow-up periods. As multidomain interventions are implemented more broadly at the population level, it will become possible to assess their impact on community-wide dementia incidence and prevalence. Looking ahead, early detection and timely intervention are expected to become as central to dementia care as they are in cardiovascular disease prevention, where there is greater awareness among both healthcare providers and the public.

The Finnish Geriatric Intervention Study to Prevent Cognitive Impairment and Disability (FINGER) was the first large, long-term RCT to show that a two-year multidomain lifestyle intervention improves cognition and reduces cognitive decline in older adults at increased dementia risk. The intervention included diet, exercise, cognitive training, social activities, and vascular risk management, and led to significant benefits in global cognition, executive function, processing speed, and memory, as well as improvements in BMI, diet adherence, and physical activity (Ngandu et al., 2015[82]; Lehtisalo et al., 2017[62]).

Following the encouraging results of FINGER, 15 other multi-component intervention studies have been conducted, and additional 11 studies are ongoing or planned spanning high-, middle-, and low-income countries across all global regions (Sakurai et al., 2025[98]). While early multidomain dementia prevention trials (2015-2019) showed mixed results, most studies conducted in the 2020s have reported beneficial cognitive effects, with negative findings largely attributable to COVID-19 disruptions. Even when primary cognitive endpoints were not met, secondary and subgroup analyses consistently showed improvements in key dementia risk factors, supporting the potential of multidomain interventions to slow cognitive decline in at-risk older adults.

In 2017 the World-Wide (WW)-FINGERS network was launched seeking to harmonize multidomain dementia prevention trials, to promote international collaboration, data and knowledge sharing, and culturally adapted multidomain lifestyle intervention trials across diverse at-risk populations worldwide (Kivipelto et al., 2020[57]; Sindi et al., 2025[108]). Parallel brain health initiatives are also being implemented in primary care, community, and memory clinic settings. Digital, e-health-based interventions (e-FINGERS) are also under investigation (Loukas et al., 2023[71]; Rosenberg et al., 2024[96]).

The first results of the Protect Brain Health Through Lifestyle Intervention to Reduce Risk (POINTER) study have recently been published. In a single-blind, multicenter RCT of 2,111 US adults aged 60-79 years at risk of cognitive decline, a 2-year structured multidomain lifestyle intervention produced a significantly greater improvement in global cognition than a self-guided intervention (Baker et al., 2025[4]), supporting the effectiveness of higher-intensity nonpharmacological approaches for dementia prevention.

Recently, anti-amyloid monoclonal antibodies such as lecanemab and donanemab have become available for individuals with early Alzheimer's disease. However, these disease-modifying therapies are not indicated for all types of dementia, and many patients may not meet eligibility criteria. Their use also entails intensive safety monitoring, substantial costs, and other treatment burdens (Fox et al., 2025[30]; Frisoni et al., 2025[31]). Consequently, the need for effective dementia prevention strategies is increasingly urgent, given their low cost, favorable safety profile, and broad benefits for overall health and quality of life.

## 6. Conclusions

The growing evidence presented indicates that behaviors such as sustained social and cognitive engagement, adequate sleep, smoking cessation, moderation of alcohol intake, regular physical activity, and healthy dietary patterns are generally associated with better cognitive outcomes and lower dementia risk. These lifestyle factors appear to influence multiple biological pathways implicated in neurodegeneration, including vascular health, systemic and neuroinflammation, metabolic regulation, and neuroplasticity, and they often exert cumulative effects across the life course. For example, regular physical activity can enhance cerebral blood flow and promote synaptic plasticity, while healthy dietary patterns such as the Mediterranean or MIND diets may reduce oxidative stress and support neuronal function. Similarly, maintaining social and cognitive engagement has been linked to increased cognitive reserve, potentially delaying the onset of clinical symptoms even in the presence of neuropathology.

Although causal inference is constrained by methodological challenges-such as confounding, reverse causation, and the limited availability of long-term RCTs-the overall consistency of observational evidence underscores the potential benefits of a multidomain approach. Integrating several lifestyle factors simultaneously may yield additive or synergistic effects, enhancing resilience against dementia-related pathophysiology. Importantly, these interventions are not only feasible and low-risk but also adaptable, offering a population-level strategy for dementia prevention. Future research should focus on long-term, pragmatic trials that examine the timing, intensity, and combination of lifestyle interventions, further explore interactions with genetic risk factors such as APOE ε4, and evaluate effects on cognitive trajectories, functional outcomes, and underlying biomarkers. By advancing such knowledge, it may become possible to design personalized, culturally adapted prevention strategies that maximize cognitive health across diverse populations and stages of life.

## Declaration

### Funding

No funding was received for conducting the present review and for the preparation of this manuscript.

### Conflict of interest

The authors declare no conflict of interest.

### Artificial Intelligence (AI) - assisted technology

Artificial intelligence tools were used exclusively to support language editing, text organization, and summarization. All scientific content, interpretations, and conclusions were reviewed and validated by the authors, who retains full responsibility for the final manuscript.

## Figures and Tables

**Table 1:**
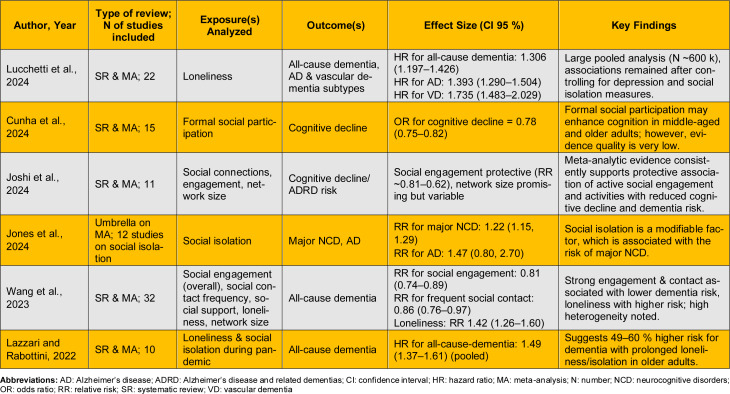
Summary of the main findings of recent meta-analyses that evaluated associations of social engagement, social isolation/loneliness, and the risk of cognitive decline or dementia risk

**Table 2:**
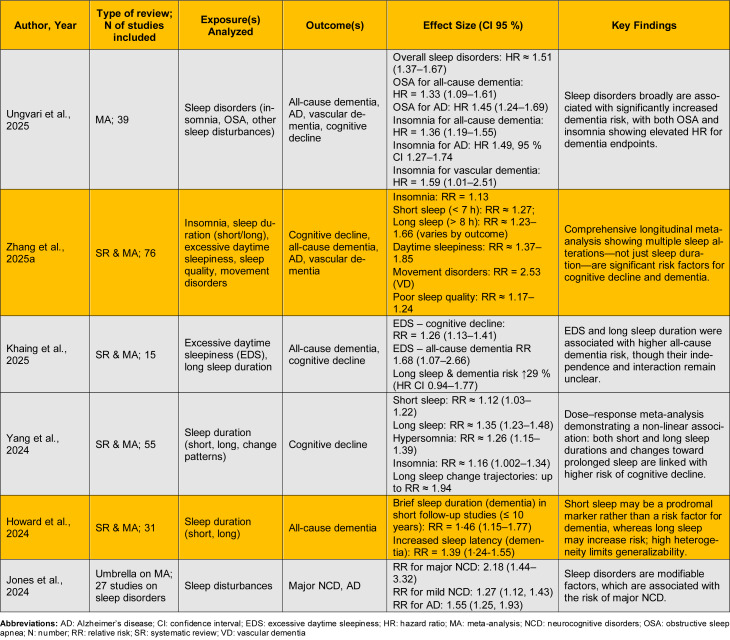
Summary of the main findings of recent meta-analyses that evaluated associations of sleep disorders and the risk of cognitive decline or dementia risk

**Table 3:**
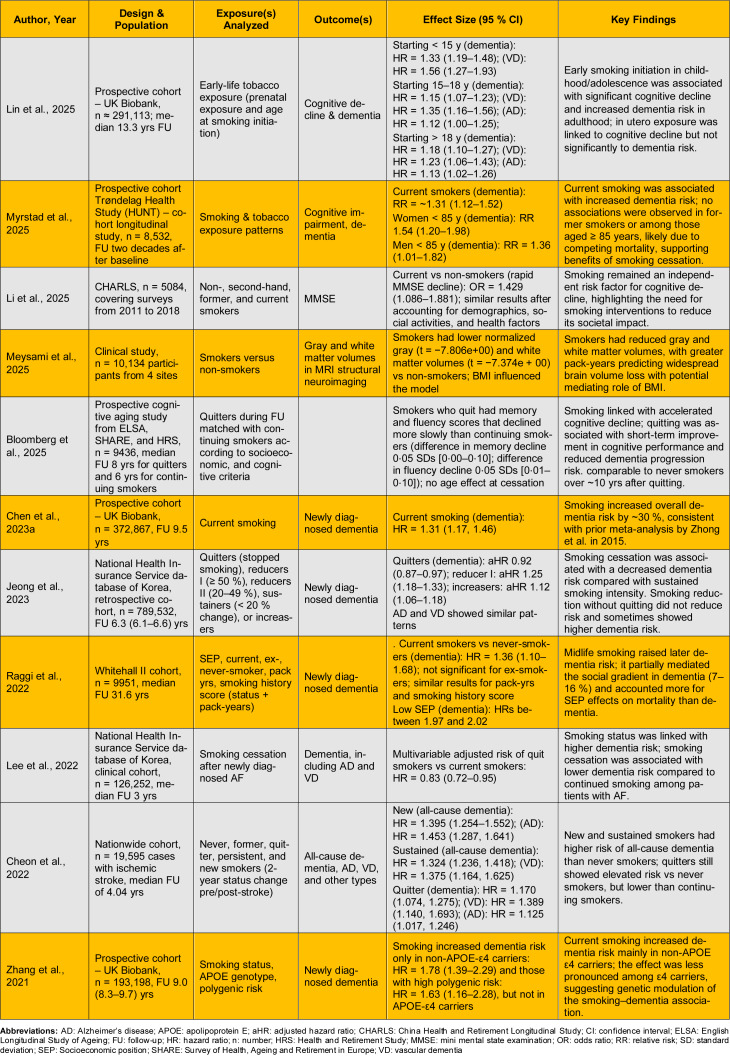
Summary of the main findings of recent cohort studies that evaluated associations of smoking and the risk of cognitive decline or dementia risk

**Table 4:**
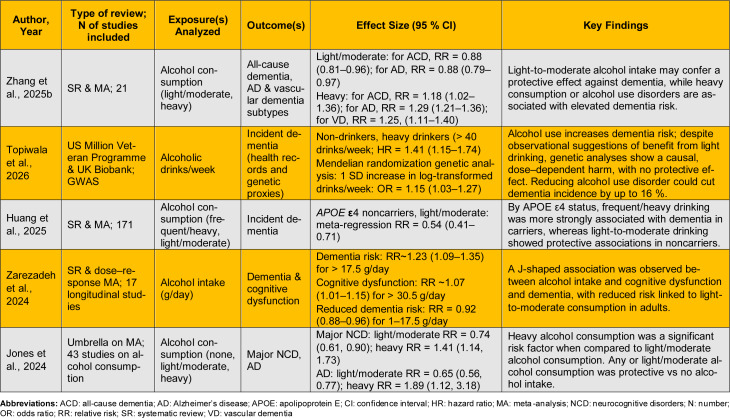
Summary of the main findings of recent meta-analyses and large-scale cohorts that evaluated associations of excessive alcohol consumption and the risk of cognitive decline or dementia risk

**Table 5:**
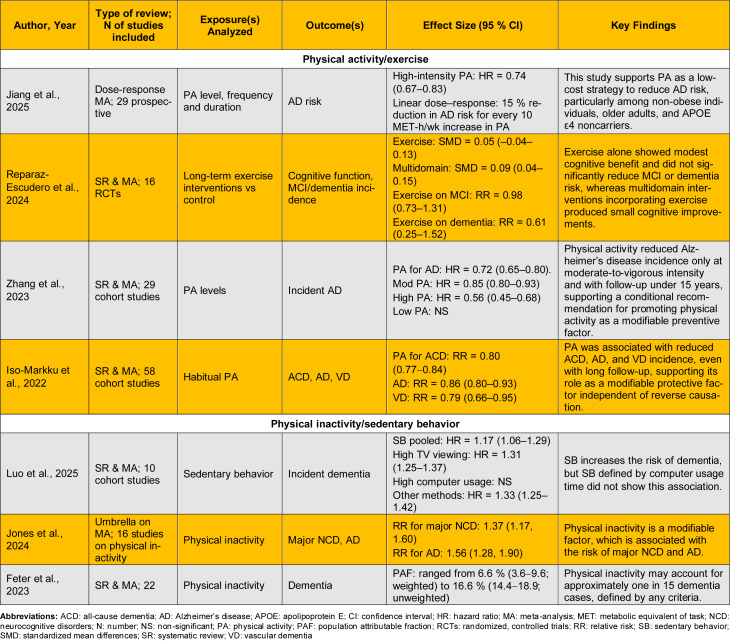
Summary of the main findings of recent meta-analyses that evaluated physical exercise and the risk of cognitive decline or dementia risk

**Table 6:**
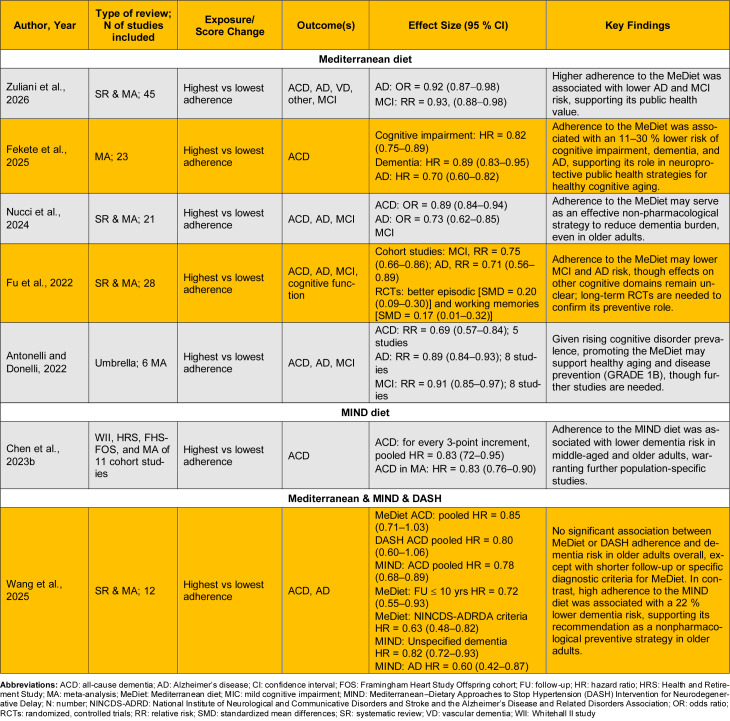
Summary of the main findings of recent meta-analyses that evaluated adherence to Mediterranean, MIND, and DASH diets and the risk of cognitive decline or dementia risk

**Figure 1:**
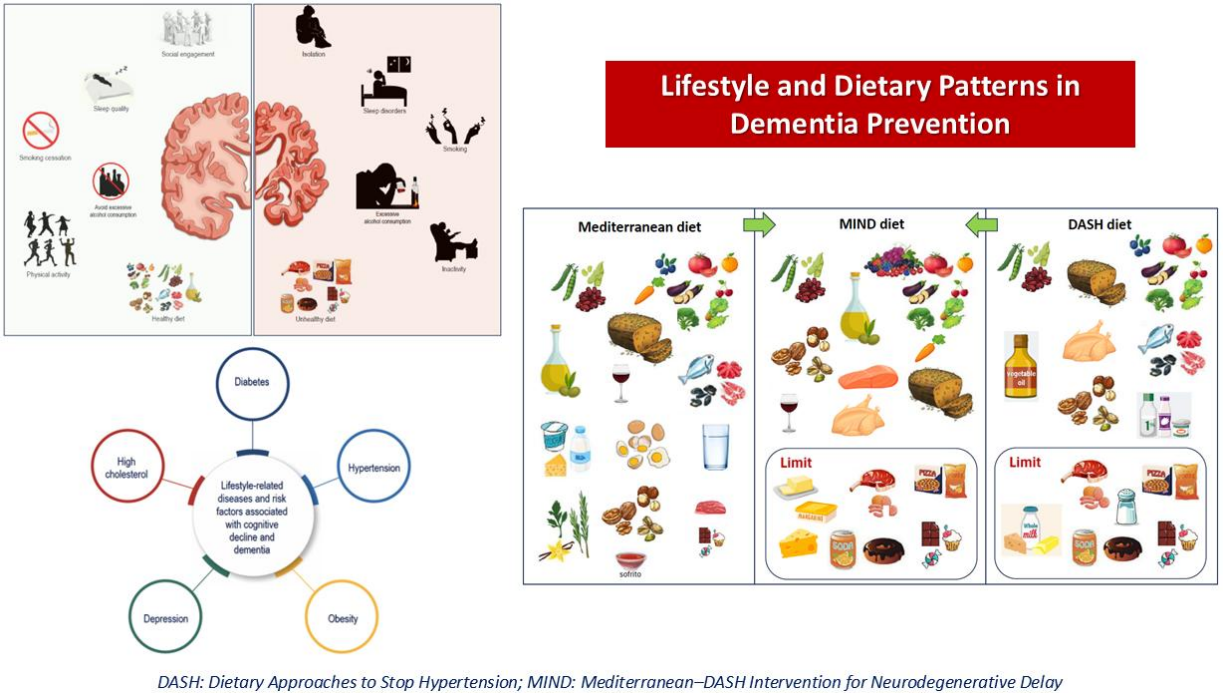
Graphical abstract
